# Cases in Practice

**Published:** 1857-09

**Authors:** E. K. Bowman

**Affiliations:** Rock Island


### ARTICLE III.—*Cases in Practice.* By E. K. Bowmam, M. D., of Rock Island.

### CASE I.—OBSTETRICAL.

During the winter of 1848 and 49, known as the winter of the deep snow. I was called out of my bed to visit a woman in labor. The midwives, two of them had broken down. I had been exposed to intense cold all day and had had all the fingers on both hands frozen—had only been home long enough to eat my supper, get to bed and get warm, when the messenger arrived. After thinking a moment to see if I could not get up some good excuse for declining, and failing to satisfy myself, I rolled out and waded my horse five miles through the drifted snow. I found the woman in terrific labor, all hands crying. Although nearly frozen I was importuned with startling vehemence not to lose a moment, accordingly I lost not a moment in making an examination, and found the childs head pressing tremendously against the external parts, but the exit from the vagina closed by a heavy septum one fourth of an inch thick and of cartilaginous firmness; a small aperture immediately under the meatus urinarius with difficulty admitted the point of the index finger. Dilatation was as impossible as with so much sole leather.—Shivering with cold worse than a man in ague, I was compelled to imbibe a cup of hot coffee, kindly held to my lips by a lady friend, while my hands were engaged in dilating efforts. Utterly failing in these efforts I determined to resort to the knife. After explaining to the husband and mother of the woman the danger of rupture of the perinæum from the sudden passage of the childs head under such powerful expulsive efforts, I resorted to the knife, and while the midwife held the candle, with one sweep of the probe pointed bistoury relieved the obstruction.—Supporting the perinæum, no injury was sustained by that part. The couple had lived together seven years quietly and apparently happy, in the vigor of youth but debarred by this barrier from sexual enjoyment. Modesty prevented them from confiding in any older person until *accidental* conception, and then bistoury forever demolished the formidable barrier. I have since delivered the woman of healthy children and no difficulty was found whatever.

### CASE II.—DEFORMITY OF VAGINA FROM ULCERATION CAUSED BY INJURY DURING PARTURITION.

On the night of the 25th ult., I was called to see an obstetrical case in Mercer county, near Camp Creek. I went with great reluctance as the condition of the roads made them almost impassable, the night almost of pitchy darkness added to the difficulties of the way. On arriving I found the woman in labor. A brother physician was present and had been in attendance most of the day, and had sent for me having encountered as he supposed more difficulty than he was willing to take the responsibility of attempting to solve alone. A careful examination disclosed the following condition of the parts: The meatus urinarius was dilated and tender from the forcible insertion of finger, the urine dribling away involuntarily, the sphincter from excessive dilation having lost its contractility. The vagina was closed from the superior commissure to the inferior by a thick firm cicatrix, save at the lower and left side, where an aperture was found so small that the woman told me she had been only able to introduce a common wooden pencil, and that caused pain and made the part bleed. The woman was married to her second husband, had had a child by her former husband, and injury by the midwife at the time of her first confinement, followed by inflammatory action, had caused the adhesion of the walls of the vagina. Under such circumstances she had married again. Of course the gate was effectually closed on all natural sexual communication with the second husband. Yet nature asserted her rights, and notwithsanding such an apparently impenetrable barrier had received the impregnating fluid and conception took place. The pains being regular and natural, I advised waiting until the head of the foetus emerging from the inferior straits of the pelvis should forcibly rest against the external parts. In about an hour this occurred (12 o'clock at night.) Then after placing the woman in convenient position, scalpel in hand, my brother acoucheur assisting with the light, I proceeded with every occurrence of the expulsive effort carefully to dissect longitudinally along the center of the line of the cicatrix, and ceasing to cut the moment the pain relaxed succeeded after cutting $\frac{3}{8}$ of an inch in depth $1\frac{1}{2}$ inches long in reaching the head of the foetus. Careful dilatation with the finger and support of the perinœum co-operating with the efforts of nature, accomplished delivery safely in a short time. The child was vigorous and large, and promises to do no discredit to the race of W—— Tell to which he belongs. This case proves conclusively that conception may take place when the accomplishment of the sexual act is physically impossible.

CASE III.—POISONING BY STRYCHNINE.

In the summer of 1850, while eating my breakfast I was interrupted by a young man in great alarm, who wished my immediate presence at the sulphur springs, as a comrade had just taken quinine to break ague, set down to his breakfast and commenced to drink a cup of coffee: while drinking he fell over from the table in a terriffic fit as they called it. In a few minutes I was there, found the young man in a fearful spasm, the spine was curved backwards so as to resemble the concavity of a large hoop, the eyes staring and protuberant and muscles as rigid as iron. A moments observation flashed conviction through my mind that the subject before me was under the influence of strychnine. Acting on this decision I at once proceeded with the energy of desperation. I had nearly four ounces of ipecac in my pill bags, and at once gave him a teacup full of a mixture of it. I then set my scarrificator to its utmost depth and it having a powerful spring sent the 16 blades well home. I placed cups and common tumblers until I had the whole spine covered with them. Then drenched him with ipecac until I had given my whole supply yet no vomiting occurred—renewed the cups and resorted to tartar emetic, six grain doses, every ten or fifteen minutes. Plying the cups the spasms abated, he soon began to relax and presently heaved "like a dog with a bone in this throat." I kept him puking until bilious matter was ejected freely, and using so much warm water as to ensure a very clean condition of the unfortunate organ if water would do any good. When no more bilious matter was ejected I desisted Then returned home and procured some iodide of iron and gave him of that, having seen it recommended as being nearer an antidote for that terrific poison strychnine than anything else known. He slowly regained his health and the ague was about as effectually broken up as I ever saw it done. I will here state the manner in which the accident occurred. When the family moved into the house they found a common, long, one ounce vial, having about as much of a light white powder in it as would equal in bulk ten grains of old quinine. It was brought out into the road to me one day to know if it was not quinine, carelessly looking at it, knowing that the family that had been living there before, had had ague and treated themselves for it, I supposed it quinine and replied I guessed it was —not dreaming of finding strychnine in such quantity and in such a bottle and no label on it. The mistake was natural, but I then for the first and last time, deviated from my rule always to advise all old medicines to be committed to the flames.

